# Supplementary material for: Ceramic-like stable CsPbBr3 nanocrystals encapsulated in silica derived from molecular sieve templates
Source: Nat Commun. 2020 Jan 7;11:31. doi: 10.1038/s41467-019-13881-0 (PMC6946649; doi:10.1038/s41467-019-13881-0)
Supplement: Supplementary file 1 — Supplementary Information [file 41467_2019_13881_MOESM1_ESM.pdf]

Supplementary Information

# Ceramic-like stable $CsPbBr_3$ nanocrystals encapsulated in silica derived from molecular sieve templates

Zhang et al.

## Supplementary Methods

**Preparation of colloidal $CsPbBr_3$ NCs**. Colloidal $CsPbBr_3$ NCs were synthesized according to our previous work[1]. 10 mmol $Cs_2CO_3$, 20 mL ODE, and 20 mL OA were added into a 100 mL three-neck flask and evacuated for 1 h at 120 ℃ under standard air free conditions. Then, the temperature was raised to 150 ℃ under argon flow to form clear solution. After 0.5 h, the solution was cooled down to room temperature, and stored as stock solution (0.5 M, CsOA precursor).

2 mmol $PbBr_2$, 20 mL ODE, 5 mL OAm, 5 mL OA were added into a 100 mL three-neck flask and evacuated for 30 min at 120 ℃. Then, the temperature raised to 180 ℃ under argon atmosphere until $PbBr_2$ salt had completely dissolved. Afterward, 1 mL of CsOA precursor (0.5 M), which was pre-heated at 70 ℃, was injected into the prepared solution. After 10 s, the three-neck flask was placed in an ice-water bath and cooled to room temperature. Equal methyl acetate was added into the crude solution and precipitated via centrifugation at 10000 rpm. The precipitate was dispersed in 30 mL of toluene solution.

**Preparation of CdSe/CdS/ZnS NCs**. CdSe/CdS/ZnS core/shell NCs were synthesized according to our previous work[2].

## Supplementary Figures, Supplementary Tables and Supplementary Discussion

Supplementary Table 1. Properties and identification information of molecular sieves.

| Common name | MCM-41 | ZSM-5 | NaY | Y-Zeolite |
|---|---|---|---|---|
| Pore size, nm | 3.6 | 0.5 | 1.0 | 3.5 |
| Surface area,$m^2$ $g^{-1}$ | 1037 | 380 | 850 | 650 |
| Named | MS | ZSM | NaY | Y-Zeolite |

Supplementary Table 2. The nominal compositions of control groups.

| CsBr | $PbBr_2$ | Mass ratio ($CsBr/PbBr_2$):MS | MS/mg | Calcination temperature/℃ | Named |
|---|---|---|---|---|---|
| 0.6mmol, 127.7mg | 0.6mmol, 221.4mg | 1:3 | 1047.3 | 400 | $CsPbBr_3$-$SiO_2$-400 |
| 0.6mmol, 127.7mg | 0.6mmol, 221.4mg | 1:3 | 1047.3 | 500 | $CsPbBr_3$-$SiO_2$-500 |
| 0.6 mmol, 127.7mg | 0.6mmol, 221.4mg | 1:3 | 1047.3 | 600 | $CsPbBr_3$-$SiO_2$-600 |
| 0.6 mmol, 127.7mg | 0.6mmol, 221.4mg | 1:3 | 1047.3 | 700 | $CsPbBr_3$-$SiO_2$-700 |
| 0.6 mmol, 127.7mg | 0.6mmol, 221.4mg | 1:3 | 1047.3 | 800 | $CsPbBr_3$-$SiO_2$-800 |
| 0.6 mmol, 127.7mg | 0.6mmol, 221.4mg | 1:3 | 1047.3 | 900 | $CsPbBr_3$-$SiO_2$-900 |
| 4 mmol, 851.3mg | 4mmol, 1476.1mg | 10:1 | 232.7 | 700 | $CsPbBr_3$-$SiO_2$ (10:1) |
| 4 mmol, 851.3mg | 4mmol, 1476.1mg | 5:1 | 465.5 | 700 | $CsPbBr_3$-$SiO_2$ (5:1) |
| 4 mmol, 851.3mg | 4mmol, 1476.1mg | 3:1 | 775.8 | 700 | $CsPbBr_3$-$SiO_2$ (3:1) |
| 0.6 mmol, 127.7mg | 0.6mmol, 221.4mg | 1:1 | 349.1 | 700 | $CsPbBr_3$-$SiO_2$ (1:1) |
| 0.6 mmol, 127.7mg | 0.6mmol, 221.4mg | 1:3 | 1047.3 | 700 | $CsPbBr_3$-$SiO_2$ (1:3) |
| 0.6 mmol, 127.7mg | 0.6mmol, 221.4mg | 1:5 | 1745.5 | 700 | $CsPbBr_3$-$SiO_2$ (1:5) |
| 0.6 mmol, 127.7mg | 0.6mmol, 221.4mg | 1:10 | 3491.0 | 700 | $CsPbBr_3$-$SiO_2$ (1:10) |

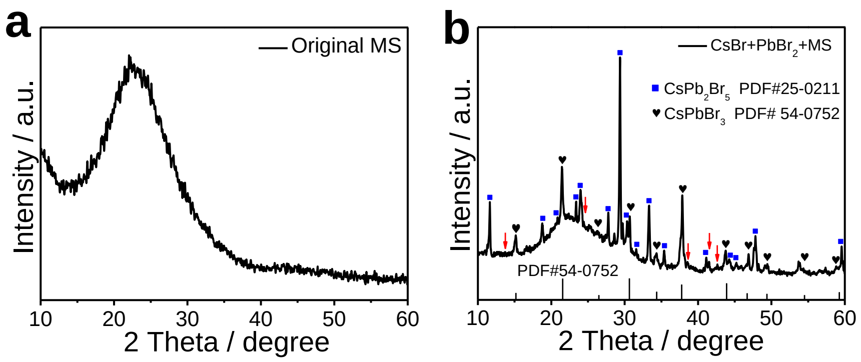


Supplementary Figure 1. a, XRD pattern of original MS. b, XRD pattern of the obtained mixture of $CsBr$, $PbBr_2$ and MS after the evaporation of water without calcination.

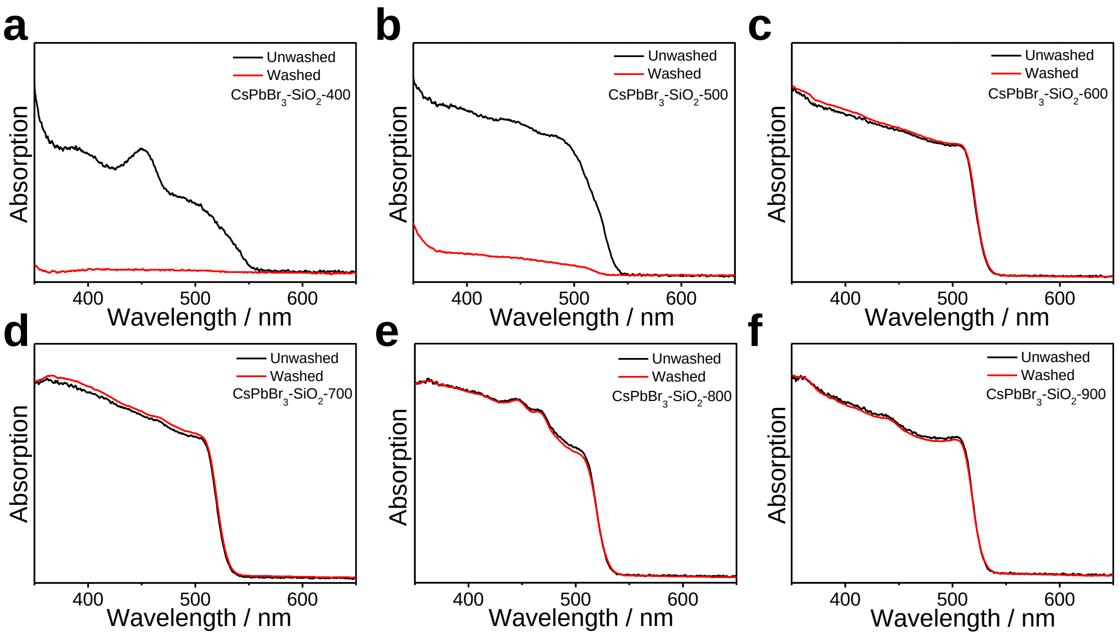


Supplementary Figure 2. UV-Vis absorption spectra of $CsPbBr_3$-$SiO_2$ powders before and after water washing: (a) 400 °C, (b) 500 °C, (c) 600 °C, (d) 700 °C, (e) 800 °C, (f) 900 °C.

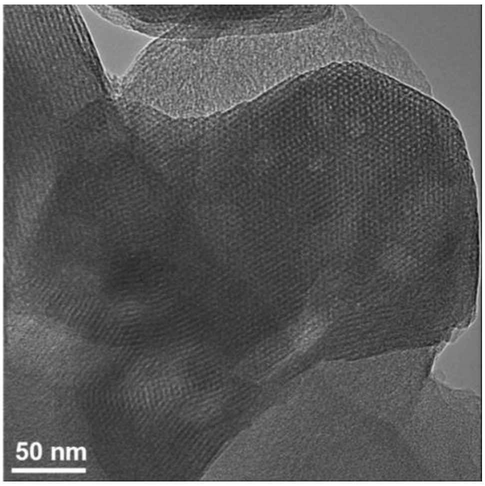


Supplementary Figure 3. TEM image of original MS.

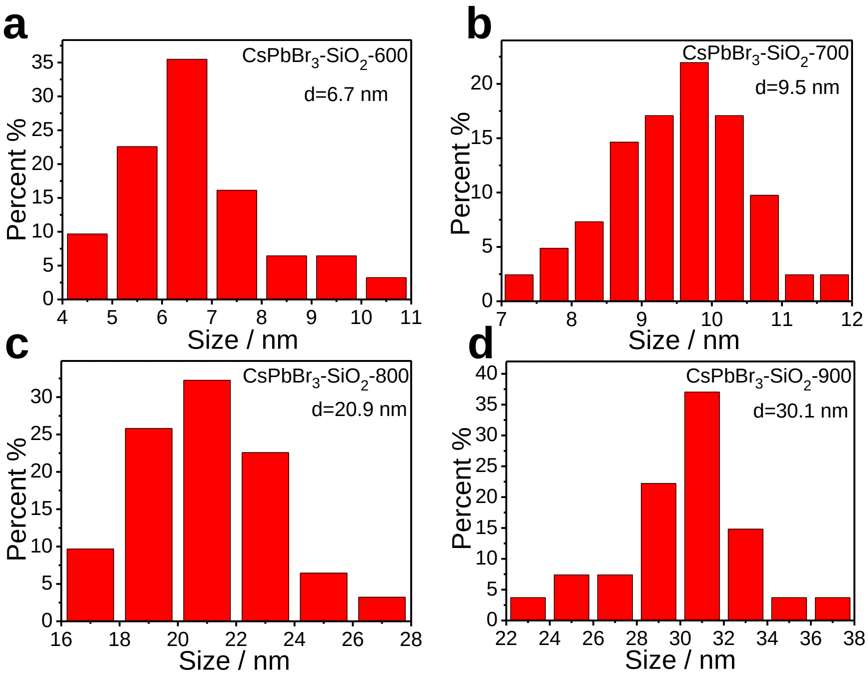


Supplementary Figure 4. The size distributions of $CsPbBr_3$ NCs ($CsBr/PbBr_2$: MS=1:3) calcined at different temperatures: (a) 600 °C, (b) 700 °C, (c) 800 °C, (d) 900 °C.

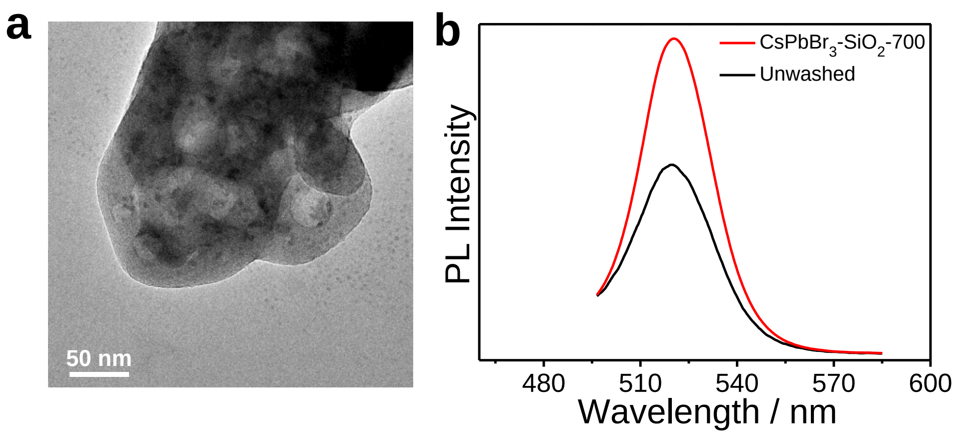


Supplementary Figure 5. a, TEM image of unwashed $CsPbBr_3$-$SiO_2$-700. b, PL spectra of $CsPbBr_3$-$SiO_2$-700 before and after water washing, excitation wavelength is 455 nm.

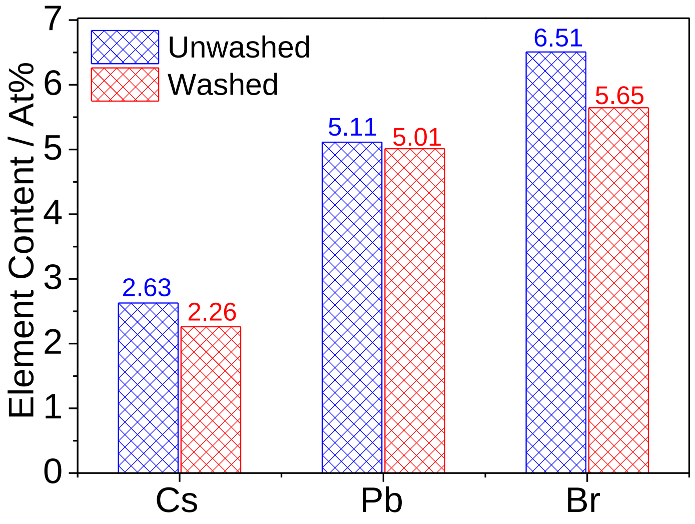


Supplementary Figure 6. Cs, Pb, and Br element contents of the unwashed and water washed $CsPbBr_3$-$SiO_2$-700 determined from XRF.

In order to further explain reaction residues and salts after water washing, X-Ray Fluorescence measurement (XRF) was used to confirm the Cs, Pb, and Br element contents of unwashed and washed $CsPbBr_3$-$SiO_2$-700. As shown in **Supplementary Figure 6**, after water washing, Cs, Pb, and Br element contents of $CsPbBr_3$-$SiO_2$-700 decreased obviously, because of removing of reaction residues and salts from the surface of $SiO_2$ that could influence the light absorption and conversion of $CsPbBr_3$ NCs in the $SiO_2$.

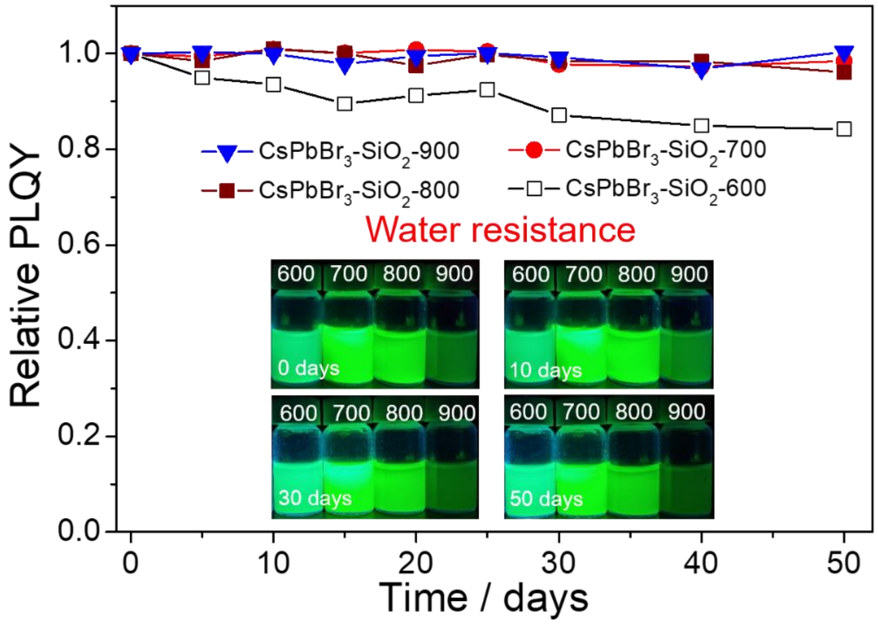


Supplementary Figure 7. Relative PLQYs and photographs (insert) of $CsPbBr_3$-$SiO_2$ (Mass ratio of $CsPbBr_3$: MS is 1:3) at different calcination temperatures immersed in water for various times.

Supplementary Table 3. The absolute PLQYs of different samples

| **Samples** | **Absolute PLQY** |
|---|---|
| $CsPbBr_3$-$SiO_2$-700 | 63% |
| $CsPbBr_3$-$SiO_2$-HF | 71% |

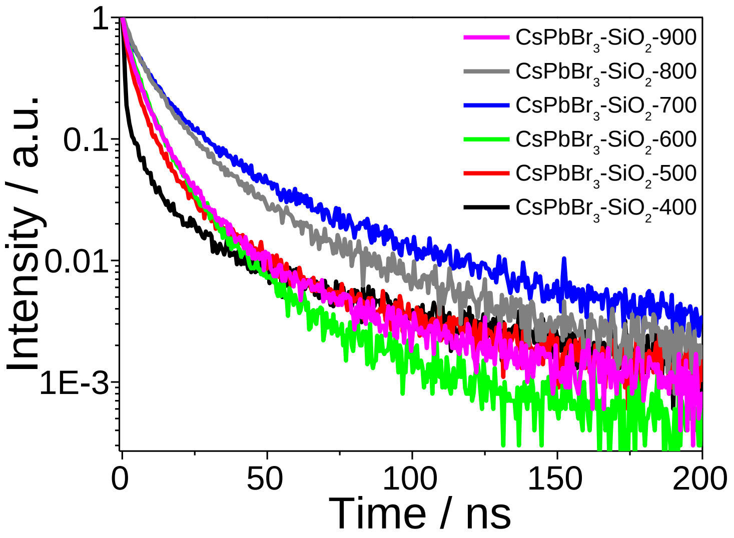


Supplementary Figure 8. Time-resolved PL decay spectra of $CsPbBr_3$-$SiO_2$ synthesized at different temperatures with the excitation wavelength at 365 nm.

Supplementary Table 4. The fitting parameters of the decay curves for $CsPbBr_3$-$SiO_2$ synthesized at different temperatures.

| Samples | $\tau_1$ (ns) | $A_1$ (%) | $\tau_2$ (ns) | $A_2$ (%) | $\tau_3$ (ns) | $A_3$ (%) | $\tau_{ave}$ (ns) |
|---|---|---|---|---|---|---|---|
| $CsPbBr_3$-$SiO_2$-400 | 0.78 | 95.84 | 11.36 | 4.16 | | | **4.86** |
| $CsPbBr_3$-$SiO_2$-500 | 1.70 | 77.43 | 6.24 | 20.81 | 31.17 | 1.76 | **8.67** |
| $CsPbBr_3$-$SiO_2$-600 | 1.85 | 31.55 | 6.03 | 60.23 | 19.32 | 8.22 | **9.25** |
| $CsPbBr_3$-$SiO_2$-700 | 2.84 | 38.70 | 10.21 | 49.66 | 39.25 | 11.65 | **21.81** |
| $CsPbBr_3$-$SiO_2$-800 | 3.58 | 41.07 | 11.04 | 51.47 | 38.28 | 7.46 | **17.72** |
| $CsPbBr_3$-$SiO_2$-900 | 1.69 | 43.30 | 6.52 | 50.62 | 24.20 | 6.08 | **10.61** |

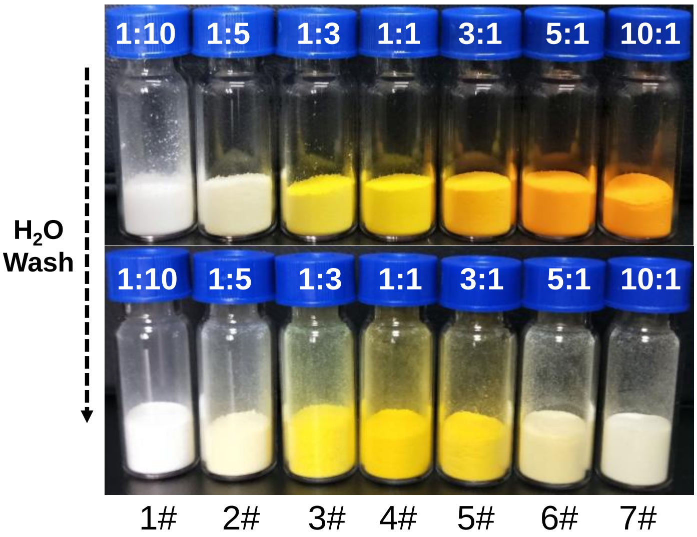


Supplementary Figure 9. Photographs of the unwashed $CsPbBr_3$-$SiO_2$ powders (upper) and water washed $CsPbBr_3$-$SiO_2$ powders (bottom) with different mass ratio of CsBr/$PbBr_2$: MS synthesized at 700 $^oC$.

1# CsBr/$PbBr_2$: MS=1:10; 2# CsBr/$PbBr_2$: MS=1:5; 3# CsBr/$PbBr_2$: MS=1:3; 4# CsBr/$PbBr_2$: MS=1:1; 5# CsBr/$PbBr_2$: MS=3:1; 6# CsBr/$PbBr_2$: MS=5:1; 7# CsBr/$PbBr_2$: MS=10:1.

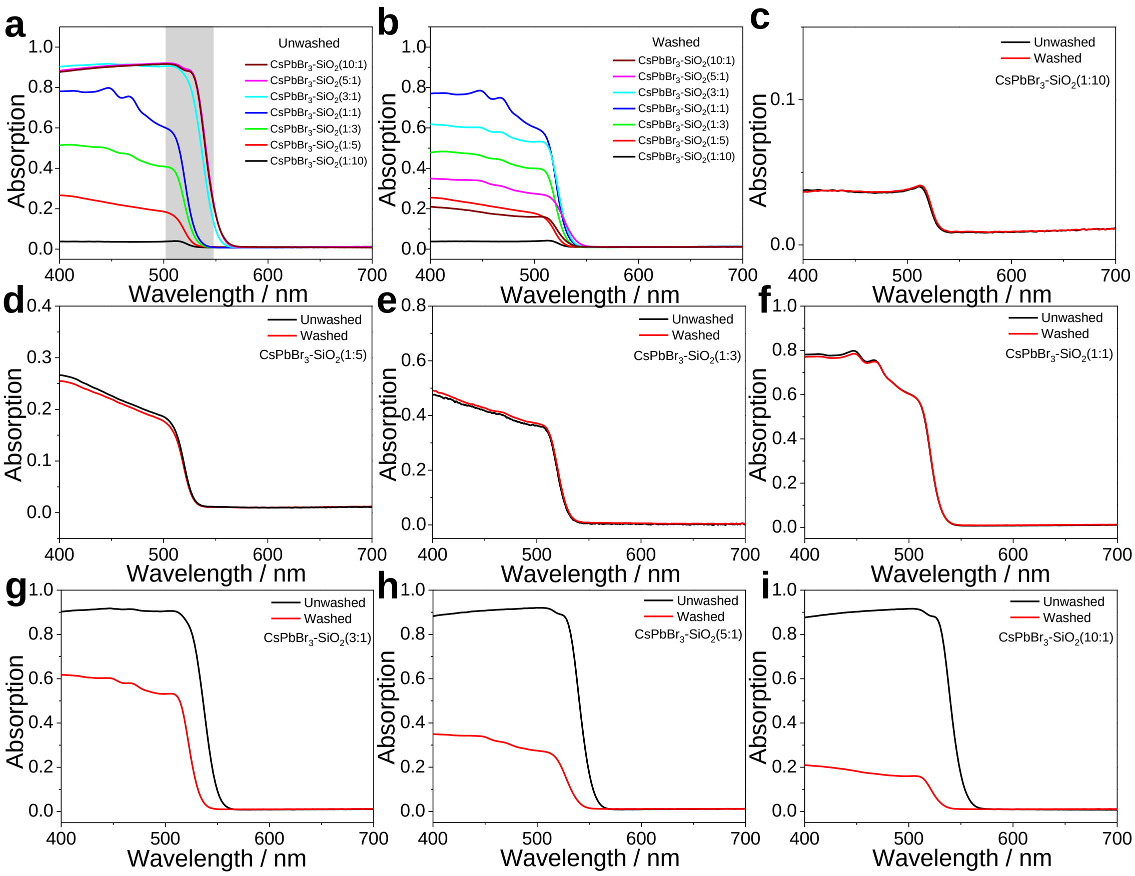


Supplementary Figure 10. UV-Vis absorption spectra of (a) unwashed $CsPbBr_3$-$SiO_2$ powders with different mass ratio of $CsBr/PbBr_2$: MS synthesized at 700 $^{o}C$, (b) water washed $CsPbBr_3$-$SiO_2$ powders with different mass ratio of $CsBr/PbBr_2$: MS synthesized at 700 $^{o}C$, (c-i) UV-Vis absorption spectra of unwashed and washed $CsPbBr_3$-$SiO_2$ powders with different mass ratio of $CsBr/PbBr_2$: MS (from 1:10 to 10:1) synthesized at 700 $^{o}C$.

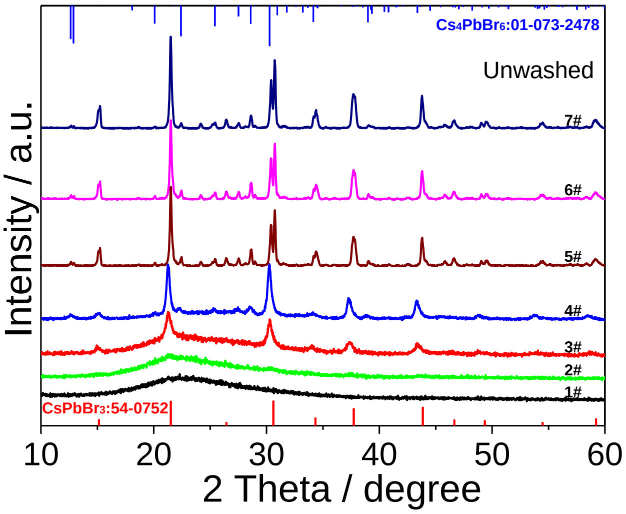


Supplementary Figure 11. XRD patterns of $CsPbBr_3$-$SiO_2$ powders with different mass ratio of $CsBr/PbBr_2$: MS at 700 °C. ($CsPbBr_3$: PDF# 54-0752; $Cs_4PbBr_6$: PDF# 01-073-2478).

1# $CsBr/PbBr_2$: MS=1:10; 2# $CsBr/PbBr_2$: MS=1:5; 3# $CsBr/PbBr_2$: MS=1:3; 4# $CsBr/PbBr_2$: MS=1:1; 5# $CsBr/PbBr_2$: MS=3:1; 6# $CsBr/PbBr_2$: MS=5:1; 7# $CsBr/PbBr_2$: MS=10:1.

**Supplementary Figure 9** illustrated the photographs of $CsPbBr_3$-$SiO_2$ with different mass ratio of $CsBr/PbBr_2$: MS synthesized at 700 °C. The colors of $CsPbBr_3$-$SiO_2$ gradually changed from white to deeper yellow with the mass ratio of $CsBr/PbBr_2$: MS increasing from 1:10 to 10:1. After water washing, the colors of $CsPbBr_3$-$SiO_2$ ($CsBr/PbBr_2$: MS=3:1, 5:1, 10:1, means excess of $CsBr/PbBr_2$) began to lighten, which indicated that a majority of $CsPbBr_3$ bulk crystals were formed on the outside of MS when employed exceedingly high mass ratio of $CsBr/PbBr_2$: MS.

Similar results can be obtained from UV-Vis absorption spectra (**Supplementary Figure 10**). Absorption intensities of $CsPbBr_3$-$SiO_2$ increased gradually with the raising of mass ratio of $CsBr/PbBr_2$: MS. After water washing, the absorption intensities of $CsPbBr_3$-$SiO_2$ with high mass ratio of $CsBr/PbBr_2$: MS (3:1, 5:1, 10:1) decreased obviously, indicating that the bulk crystals outside of MS were removed by water washing.

The XRD patterns (**Supplementary Figure 11**) confirmed the formation of cubic $CsPbBr_3$ NCs (PDF# 54-0752) with the mass ratio of $CsBr/PbBr_2$: MS=1:3, but the characteristic diffraction peaks were not observed in $CsPbBr_3$-$SiO_2$ ($CsBr/PbBr_2$: MS=1:10, 1:5), owing to the lower concentration of $CsPbBr_3$ NCs. However, except the existence of the cubic $CsPbBr_3$ NCs, we also observed the sharp diffractions from $Cs_4PbBr_6$ (PDF# 01-073-2478) in $CsPbBr_3$-$SiO_2$ with the increase of mass

ratio of $CsBr/PbBr_2$: MS from 1:1 to 10:1, which indicated that bulk crystals ($CsPbBr_3$ and $Cs_4PbBr_6$) were formed.

Therefore, excessive $CsBr/PbBr_2$ raw materials did not promote PL intensities or PLQYs of resultant $CsPbBr_3$-$SiO_2$, and the main reason can be attributed to the availability of the pores/cavities of MS that may encapsulate $CsPbBr_3$ NCs. By optimizing the mass ratio of $CsBr/PbBr_2$: MS, $CsPbBr_3$-$SiO_2$ (1:3) (t=700 $^{o}$C, mass ratio of $CsPbBr_3$: MS is 1:3) exhibited the highest absolute PLQY of 63%.

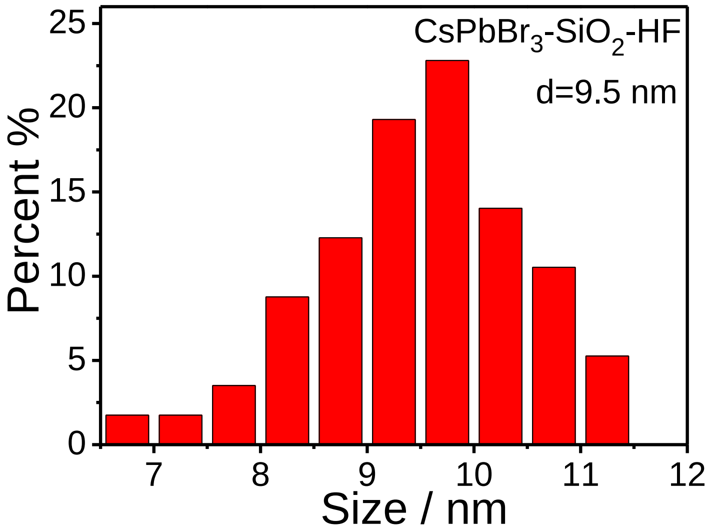


Supplementary Figure 12. The size distribution of $CsPbBr_3$ NCs from $CsPbBr_3$-$SiO_2$-HF.

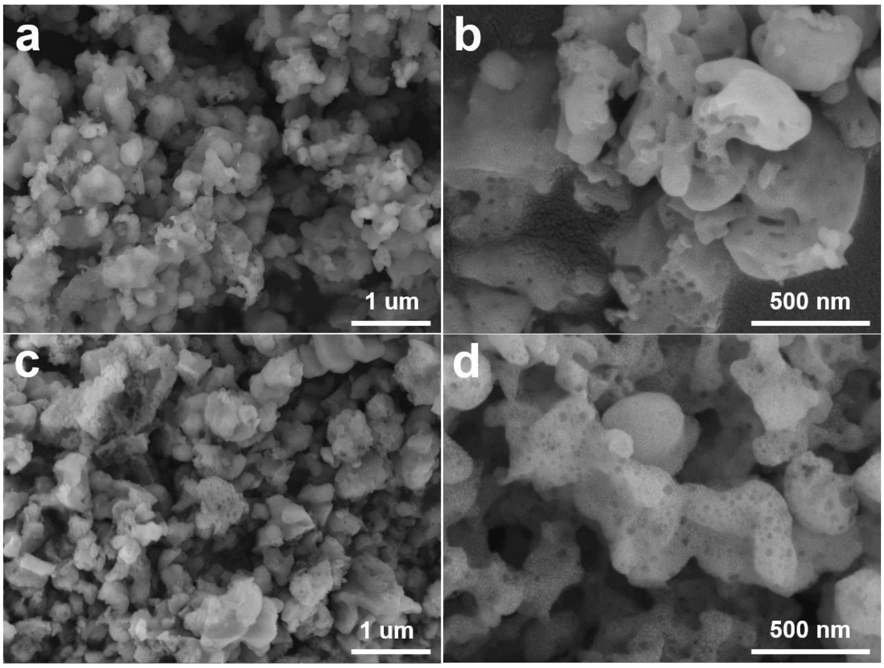

Supplementary Figure 13. SEM images of $CsPbBr_3$-$SiO_2$-700 (a, b) and $CsPbBr_3$-$SiO_2$-HF (c, d).

Supplementary Table 5. Surface area of $CsPbBr_3$-$SiO_2$-700 and $CsPbBr_3$-$SiO_2$-HF calculated with BET method.

| **Samples** | **Surface Area/ $m^2$ $g^{-1}$** |
|---|---|
| $CsPbBr_3$-$SiO_2$-700 | 9.6 |
| $CsPbBr_3$-$SiO_2$-HF | 16.7 |

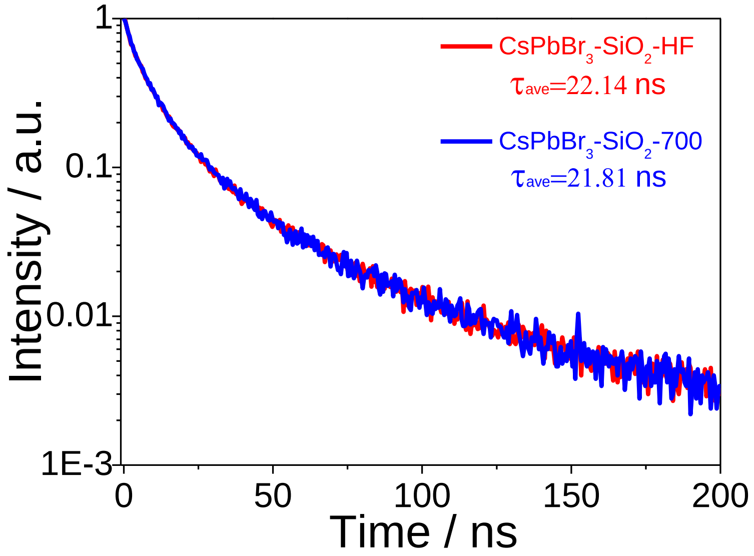


Supplementary Figure 14. Time-resolved PL decay spectra of $CsPbBr_3$-$SiO_2$-700 and $CsPbBr_3$-$SiO_2$-HF with the excitation wavelength at 365 nm.

Supplementary Table 6. The fitting parameters of the decay curves for $CsPbBr_3$-$SiO_2$-700 and $CsPbBr_3$-$SiO_2$-HF.

| Samples | $\tau_1$ (ns) | $A_1$ (%) | $\tau_2$ (ns) | $A_2$ (%) | $\tau_3$ (ns) | $A_3$ (%) | $\tau_{ave}$ (ns) |
|---|---|---|---|---|---|---|---|
| $CsPbBr_3$-$SiO_2$-700 | 2.84 | 38.70 | 10.21 | 49.66 | 39.25 | 11.65 | **21.81** |
| $CsPbBr_3$-$SiO_2$-HF | 2.84 | 38.16 | 10.08 | 50.54 | 40.06 | 11.30 | **22.14** |

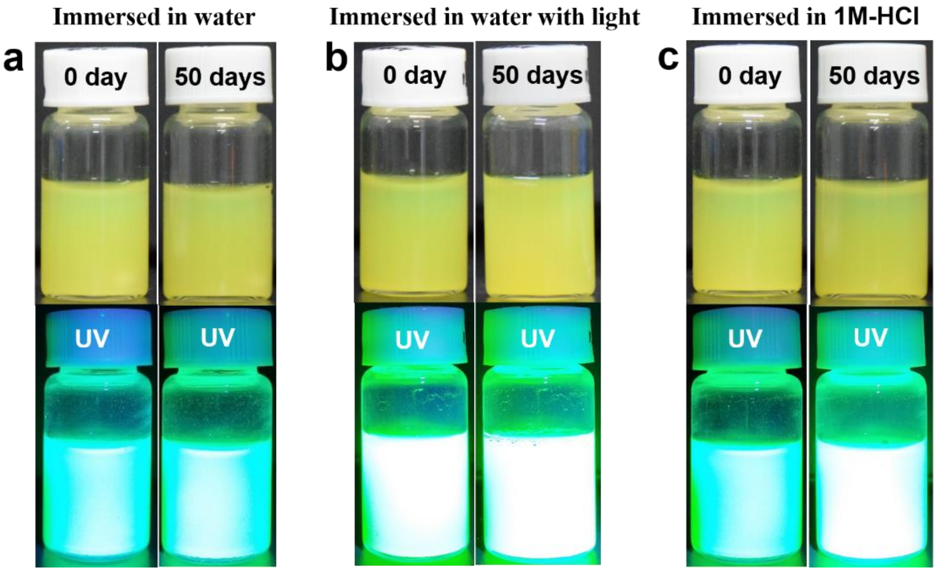


Supplementary Figure 15. Photographs of $CsPbBr_3$-$SiO_2$-700 immersed in water (a), immersed in water under illumination with a 450 nm LED light (175 mW $cm^{-2}$) (b), immersed in 1M HCl (c) for 50 days, visible illumination (upper) and UV illumination (bottom).

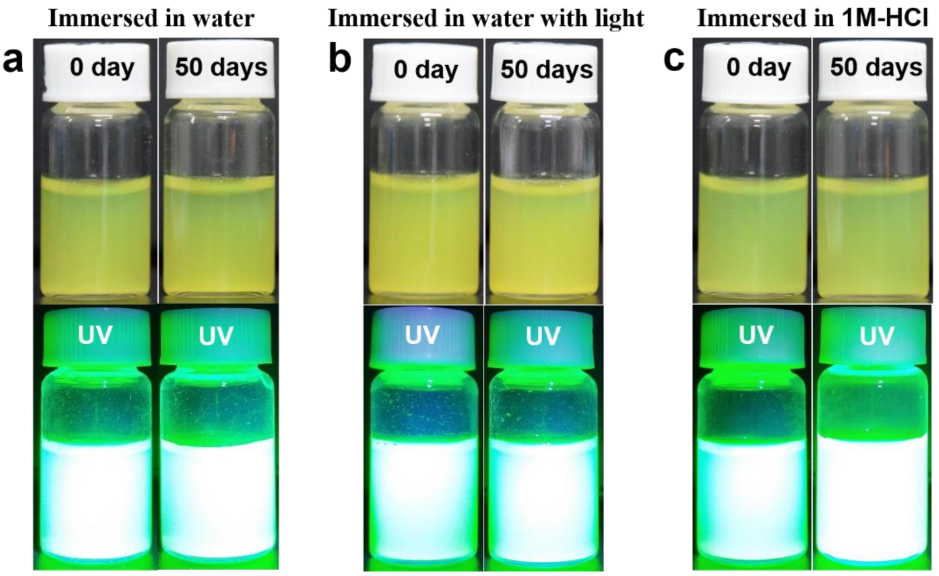


Supplementary Figure 16. Photographs of $CsPbBr_3$-$SiO_2$-HF immersed in water (a), immersed in water under illumination with a 450 nm LED light (175 mW $cm^{-2}$) (b), immersed in 1M HCl (c) for 50 days, visible illumination (upper) and UV illumination (bottom).

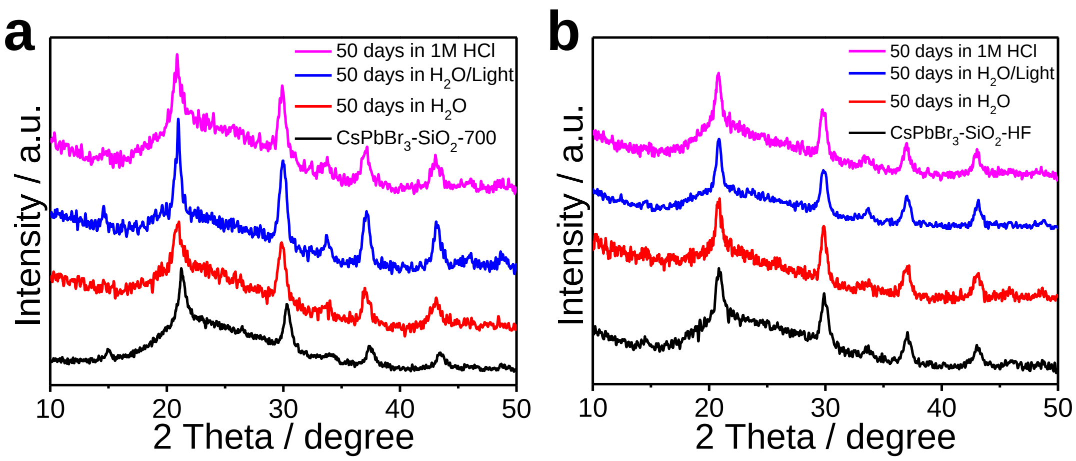


Supplementary Figure 17. XRD patterns of $CsPbBr_3$-$SiO_2$-700 (a) and $CsPbBr_3$-$SiO_2$-HF (b) after immersed in various solvents for 50 days.

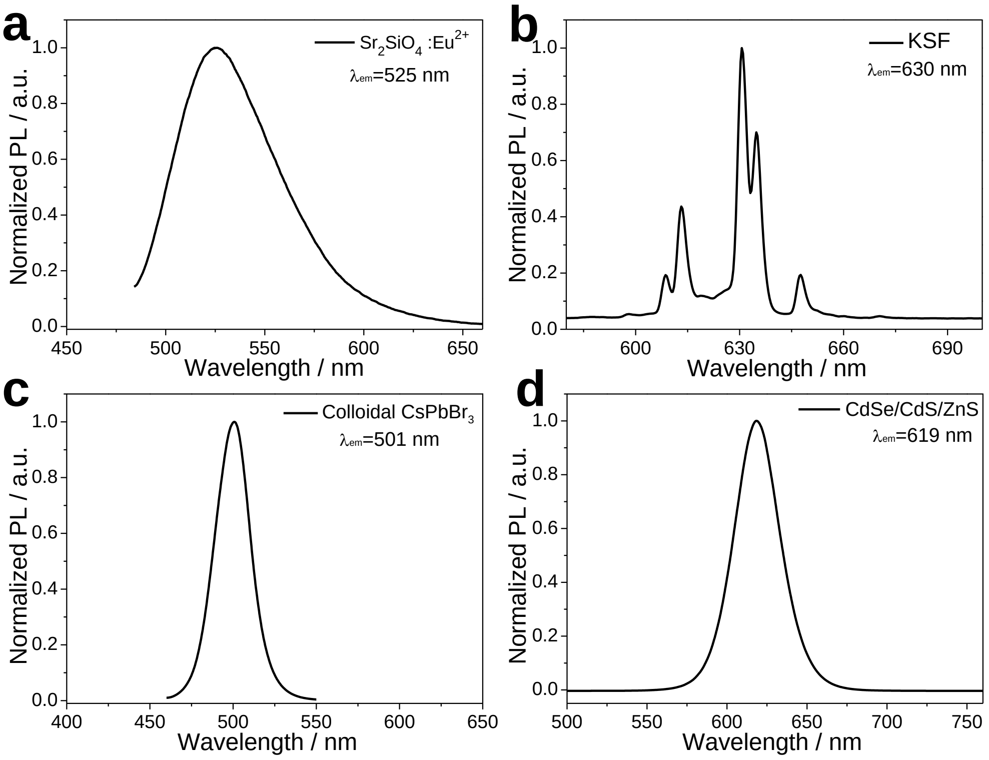


Supplementary Figure 18. Photoluminescence emission spectra of $Sr_2SiO_4:Eu^{2+}$ green phosphor (a), KSF red phosphor (b), colloidal $CsPbBr_3$ NCs (c), and CdSe/CdS/ZnS NCs (d).

Supplementary Table 7. Stability of perovskite composites by using different coating materials and methods.

| Composites | Synthetic method | PL QYs | Stability: Remnant PL | Ref. |
|---|---|---|---|---|
| $CsPbBr_3/SiO_2$ | High temperature encapsulation, MS collapse | 71% | Above 100% (1000h, illuminated with 450 nm LED light ) | This work |
| $CsPbBr_3/SiO_2$ | Hot-injection, TMOS hydrolysis | 65% | 55% (48h, under 100% RH at room temperature) | 3 |
| $CsPbX_3/SiO_2$ | Hot-injection, TEOS hydrolysis | 84% | 40% (4h, water) | 4 |
| $CsPbBr_3/SiO_2$ | Hot-injection, TMOS hydrolysis | 80% | 98% (10h, UV) | 5 |
| $CsPbBr_3/SiO_2/PM$ | Hot-injection, 3-APTES hydrolysis | 65% | 50% (60 d, stored under ambient conditions) | 6 |
| $MAPbBr_3/SiO_2$ | Hot-injection, TMOS hydrolysis | 89% | 61% ( 49h, illuminated with 450 nm LED light) | 7 |
| $CsPbBr_3/AlO_x$ | Hot-injection, Atomic Layer Deposition | 56% | 50% (8h, 100 mW $cm^{-2}$ solar irradiation) | 8 |
| $CsPbBr_3$-TDPA | Hot-injection | 68% | 80% (300min, water) | 9 |
| $CsPbBr_3/PS$ | Electrospinning technique | 48% | 70% (192h, water) | 10 |
| $CsPbX_3/PMMA$ | A microfluidic spinning technique | 45% | 75% (3 d, 30 $^{o}C$ air with 70% humidity)) | 11 |
| $CsPbX_3/CaF_2$ | Hot-injection | 82% | 60% ((2 d, air with 100% humidity) | 12 |
| $MAPbBr_3/NaNO_3$ | Reprecipitation synthesis | 42% | 80% (14h, UV) | 13 |
| $CsPbBr_3/NH_4Br$ | Hot-injection, ion-exchange | 64% | 40% (3.5h, water) | 14 |

**List of acronyms and abbreviations**

MS: molecular sieve

PM: polystyrene microspheres

TDPA: alkyl phosphate

PS: Polystyrene

PMMA: Polymethyl methacrylate

TMOS: Tetramethyl orthosilicate

TEOS: Tetraethyl orthosilicate

3-APTES: (3-amino-propyl)triethoxysilane

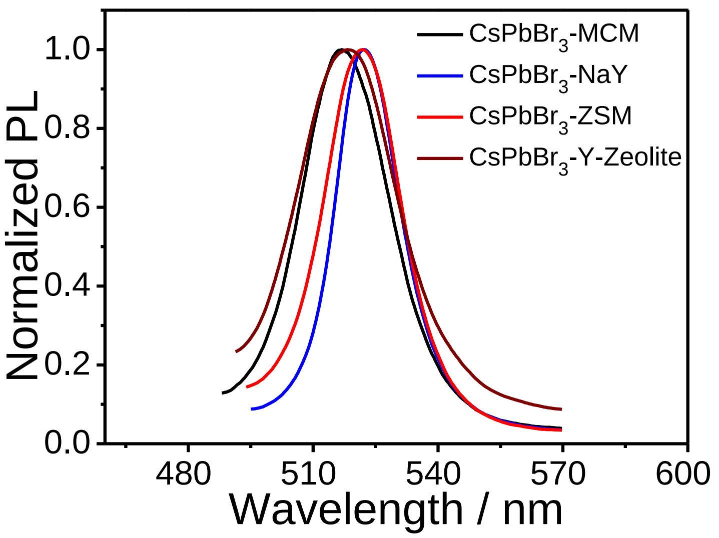


Supplementary Figure 19. PL spectra of $CsPbBr_3$ NCs in different MS, such as ZSM, NaY, Y-Zeolite, and MCM, excitation wavelength is 455 nm.

## Supplementary References


1. Li, Z., Kong, L., Huang, S. & Li, L. Highly Luminescent and Ultrastable $CsPbBr_3$ Perovskite Quantum Dots Incorporated into a Silica/Alumina Monolith. *Angew. Chem. Int. Ed.* **129**, 8246-8250 (2017).

2. Li, Z., Kong, L., Sun, H., Huang, S. & Li, L. Effect of Electronic Structure on the Stability of CdSe/CdS and CdSe/CdS/ZnS Quantum Dot Phosphor Incorporated into Silica/Alumina Monolith. *ACS Applied Nano Materials.* **1**, 3086-3090 (2018).

3. Li, L., et al. Sustainable and Self-Enhanced Electrochemiluminescent Ternary Suprastructures Derived from $CsPbBr_3$ Perovskite Quantum Dots. *Adv. Funct. Mater.* **29**, 1902533 (2019).

4. Ding, N., et al. Highly stable and water-soluble monodisperse $CsPbX_3/SiO_2$ nanocomposites for white-LED and cells imaging. *Nanotechnology* **29**, 345703 (2018).

5. Hu, H., et al. Interfacial synthesis of highly stable $CsPbX_3$/oxide Janus nanoparticles. *J. Am. Chem. Soc*. **140**, 406-412 (2017).

6. Liang, X., Chen, M., Wang, Q., Guo, S. & Yang, H. Ethanol-Precipitable, Silica-Passivated Perovskite Nanocrystals Incorporated into Polystyrene Microspheres for Long-Term Storage and Reusage. *Angew. Chem. Int. Ed.* **131**, 2825-2829 (2019).

7. Huang, S., et al. Enhancing the stability of $CH_3NH_3PbBr_3$ quantum dots by embedding in silica spheres derived from tetramethyl orthosilicate in “waterless” toluene. *J. Am. Chem. Soc*. **138**, 5749-5752 (2016).

8. Loiudice, A., Saris, S., Oveisi, E., Alexander, D.T.L. & Buonsanti, R. $CsPbBr_3$ QD/AlOx inorganic nanocomposites with exceptional stability in water, light and heat. *Angew. Chem. Int. Ed.* **56**, 10696-10701 (2017).

9. Xuan, T., et al. Highly stable $CsPbBr_3$ quantum dots coated with alkyl phosphate for white light-emitting diodes. *Nanoscale* **9**, 15286-15290 (2017).

10. Liao, H., et al. A General Strategy for In Situ Growth of All-Inorganic $CsPbX_3$ (X= Br, I, and Cl) Perovskite Nanocrystals in Polymer Fibers toward Significantly Enhanced Water/Thermal Stabilities. *Adv. Opt. Mater*. **6**, 1800346 (2018).

11. Ma, K., Du, X.-Y., Zhang, Y.-W. & Chen, S. In situ fabrication of halide perovskite nanocrystals embedded in polymer composites via microfluidic spinning microreactors. *J. Mater. Chem. C.* **5**, 9398-9404 (2017).

12. Wei, Y., et al. Highly Luminescent Lead Halide Perovskite Quantum Dots in Hierarchical $CaF_2$ Matrices with Enhanced Stability as Phosphors for White Light-Emitting Diodes. Adv. Opt. Mater. **6**, 1701343 (2018).

13. Yang, G., Fan, Q., Chen, B., Zhou, Q. & Zhong, H. Reprecipitation synthesis of luminescent $CH_3NH_3PbBr_3/NaNO_3$ nanocomposites with enhanced stability. J. Mater. Chem. C. **4**, 11387-11391 (2016).

14. Lou, S., et al. Nanocomposites of $CsPbBr_3$ perovskite nanocrystals in an ammonium bromide framework with enhanced stability. J. Mater. Chem. C. **5**, 7431-7435 (2017).
